# Cervical cancer screening in rural Ethiopia: a cross- sectional knowledge, attitude and practice study

**DOI:** 10.1186/s12885-020-07060-4

**Published:** 2020-06-17

**Authors:** Friederike Ruddies, Muluken Gizaw, Brhanu Teka, Sarah Thies, Andreas Wienke, Andreas M. Kaufmann, Tamrat Abebe, Adamu Addissie, Eva Johanna Kantelhardt

**Affiliations:** 1 Institute of Medical Epidemiology, Biometrics and Informatics, Martin-Luther-Universität Halle-Wittenberg, 06097 Halle (Saale), Germany; 2 School of Public Health, Department of Preventive Medicine, Addis Ababa University, Addis Ababa, Ethiopia; 3 Department of Microbiology, Immunology and Parasitology, Addis Ababa University, Addis Ababa, Ethiopia; 4 Clinic for Gynecology, Charité-Universitätsmedizin Berlin, corporate member of Freie Universität Berlin, Humboldt-Universität zu Berlin, and Berlin Institutes of Health, Augustenburgerplatz 1, 13353 Berlin, Germany

**Keywords:** Cervical cancer, Ethiopia, Health intervention, Acceptability, Human papillomavirus

## Abstract

**Background:**

Cervical cancer is the fourth most common cancer among women worldwide. Sub- Saharan Africa has a high incidence, prevalence and mortality due to shortage and underutilization of screening facilities. This study aims to assess knowledge and attitude towards cervical cancer and its prevention, as well as practice of cervical cancer screening.

**Methods:**

This cross-sectional community- based study was conducted in Butajira, Ethiopia in February 2018. Systematic cluster randomized sampling was used to select households from which women in the targeted age group of 30–49 years were invited to participate. Data was collected using a quantitative door to door approach. The questionnaire included socio-demographic data, obstetric history, general knowledge, risk factors, attitude and practice. Logistic regression was used to assess factors associated with knowledge, attitude and practice after dichotomizing the scores using the median as cut off point.

**Results:**

Three hundred forty-two out of 354 women completed the interviewer administered questionnaire making the response rate 96.3%. 125 women (36%) were aware of cervical cancer and 14 (4.7%) knew symptoms. None of the women named HPV as a risk factor. 61% thought it was a deadly disease, 13.5% felt at risk of developing cervical cancer and 60.7% said cervical cancer is treatable. Eight women (2.3%) had previously been screened. 48.1% had a source of information concerning cervical cancer, of which 66.5% named nurses. Better knowledge was associated with 1–8 years of education (OR = 2.4; CI: 2.4–1.3), having a source of information (OR = 9.1, CI:4.0–20.6), use of contraceptives (OR = 2.3, CI: 1.3–4.0) and a higher income (OR = 1.009, CI: 1.00–1.01). Naming nurses (OR:5.0, CI:2.4–10.3), another source of information (OR = 3.3, CI:1.2–9.0), use of contraceptives (OR = 2.2, CI:1.2–3.8) and living in an urban area (OR = 3.3, CI:1.2–9.0) were associated with a positive attitude. Naming nurses (OR = 21,0, CI:10.4–42.3) and another source of information (OR = 5.8, CI:2.4–13.5) were associated with participating in cervical cancer screening.

**Conclusion:**

Most women were unaware of cervical cancer, HPV-infection as a risk factor and did not feel susceptible to cervical cancer. As Health workers were the most commonly mentioned source of information, focus should be put on their further education.

## Background

Cervical cancer is still the fourth most common cancer among women worldwide [1]. According to the GLOBOCAN data of 2018 the incidence of cervical cancer is 563,847 new cases worldwide, of which 52,633 occur in Eastern Africa [2]. In Ethiopia incidence and mortality rates of cervical cancer are 26.4 and 18.4 / 100,000 [3, 4]. The most relevant cause of cervical cancer is a persistent infection with high risk genotypes of HPV (e.g. 16, 18, 31, 52). Other co-risk factors are smoking, a weakened immune system, multi-parity, early sexual initiation and many sexual partners, as well as a family history of cervical cancer [5]. Cervical cancer mostly develops slowly, and when detected early as precancerous lesion, it can be treated effectively. Treatment options for advanced cervical cancer are expensive and often unavailable in Ethiopia [6, 7]. In developed countries the incidence of cervical cancer has decreased due to effective screening programs [2]. Due to more pressing health issues such as HIV, TB, Malaria and gastrointestinal infections, cancer and other noncommunicable diseases were long ignored in developing countries but are rapidly becoming an issue [8, 9]. Shortage of screening facilities, financial issues, cultural factors and lack of awareness limit the uptake of cervical cancer screening in developing countries [10] such as Ethiopia [9]. In a case control study in the Tigray region, Ethiopia, lack of knowledge and low risk perception were most commonly named as reasons for no-attendance to cervical cancer screening [11]. In a qualitative study from 2012 conducted in Jimma and Addis Ababa, Ethiopia the participants named limited access, lack of awareness and financial resources, the symptomless nature of cervical cancer and the stigma associated to the disease as common barriers towards screening procedures [12]. Studies conducted in Addis Ababa, Ethiopia on HIV- positive patients identified the cost, feeling healthy, lack of awareness and fear of the test results as barriers towards cervical cancer screening [13, 14]. Fear of marital disturbance and religious reasons have also been mentioned [12, 13]. The conceptual framework of the health belief model is often used to understand determining factors for a person’s attitude and preventive health behavior [15, 16] and has been used in this study to assess the participant’s attitude. This study was carried out before starting a cervical cancer screening intervention within the same community to assess possible barriers [17]. The objective was to assess women’s knowledge, attitude and practice of cervical cancer and its screening on population level with a focus on influencing personal and cultural factors for further consideration while implementing cervical cancer screening.

## Methods

### Study design and setting

This cross-sectional community- based study was conducted in Butajira, Ethiopia in February 2018 to collect baseline information prior to a cluster-randomized trial that has been registered in clinicaltrials.gov (NCT03281135) [17]. Butajira is a district located 130 km southwest of the capital Addis Ababa in central Ethiopia with approximately 75,000 people, where the Addis Ababa University maintains a Health and Demographic Surveillance Site (HDSS) to track birth and death rates as well as migration in one urban and 9 rural Kebeles, the smallest administrative unit [18] Prior to this intervention, there were VIA- trained nurses at the general Hospital in Butajira, but no formal cervical cancer screening program. The health services in the district are organized according to the Ethiopian health sector development program with a system of health extension workers for primary health care, information distribution and community mobilization [19–21]. Health extension workers were used to reach and identify participants and distribute information.

### Participants

The WHO recommends cervical cancer screening for all women at the age of 30–49 years [22]. All women in this targeted age group who were living in the HDSS zone in Butajira and were home during the time of data collection were considered eligible. The HDSS zone in Butajira was divided into 22 clusters of 80 women each. Systematic random sampling was used to first select the household and from each household women in the targeted age group were invited to participate. The single population proportion formula was used to calculate the sample size. Sample size calculation was based on the proportion of participants who were aware of cervical cancer. This proportion of participants was assumed to be 30% based on other studies conducted in Ethiopia [9, 13, 14, 23, 24]. Most of these studies, conducted in an urban setting, stated a higher level of awareness. Since this study was conducted in a rural setting, lower awareness was assumed. 322 participants were needed to construct a 95% confidence interval with an accuracy distance from estimate to limit of the CI of 5%. The final sample size was set at 354 women to account for the expected 10% non-responders.

### Variables and operational definitions

**Knowledge** was measured with 14 questions assessing general knowledge and 15 questions asking for the perception of risk factors, with a maximum score of 35 points. **Attitude** was evaluated with 12 questions on a Likert scale from 1 to 5 with the options of “sure no, no, maybe, yes and for sure yes” to ensure understandability with a maximum of 12 points. The questions were based on the health belief model with proxy variables selected for the items susceptibility, severity, social acceptability, access, cues for actions, barriers and self-efficiency [15]. The cervical cancer screening **practice** was measured with 3 questions with a maximum of 3 points assessing screening history, screening intention and access to screening facilities (supplement 1).

Independent variables were the socio-demographic data on income, age, occupation, religion, ethnicity, marital status, residency and obstetric history. Dependent variables were the scores of knowledge, attitude and practice. The median of the score was used as a cut-off point for knowledge, attitude and practice independently [25]. Those who scored on and below the median were considered to have a bad outcome.

### Data sources /measurements

Extensive literature review was done to gather all relevant information in the field using the mesh terms cervical cancer, cervical cancer and KAP, cervical cancer Ethiopia, validity and reliability of KAP questionnaires, cervical cancer Africa, cervical cancer prevention, and cervical cancer pathology. The structure, scales and ranges of a WHO questionnaire on KAP [26] were used and adapted to the Ethiopian setting [3, 4, 9, 23, 27]. After item generation appropriate scales were selected using a nominal polytomous scale for knowledge and practice section and a bounded continuous scale for the attitude section [28]. Content validity was established by a panel of experts including a gynecologist and an epidemiologist [29]. Construct validity was tested using exploratory factor analysis.

Prior to the study, FGD were conducted in Butajira and results were used to select items. A pre-test was done to examine understandability and consistency with 30 participants in Butajira, Ethiopia in January 2018. Afterwards small changes were made to wording and scoring system of the questionnaire. The option “I don’t know” was included in the knowledge section to avoid incomplete questionnaires. The questionnaire (Additional file 1) was developed in English, translated into Amharic and back into English to check for consistency. Reliability of the questionnaire was checked by testing for internal consistency using Cronbach’s alpha [30]. Cronbach’s alpha was 0.69 for the general knowledge section, 0.847 for risk factors, and 0.737 for the attitude section.

The questionnaire consisted of 67 closed questions in 7 sections on socio-demographic data, obstetric history, general knowledge, risk factors, attitude, practice and source of information.

Before starting the study, all health extension workers and data collectors were educated on cervical cancer, symptoms, HPV and possible screening methods. In the beginning of the study data collectors were trained on the questionnaire by explaining the questions, their purpose, possible answers, as well as the skip pattern. The questionnaire, the purpose and topic of the study were explained to the participants by the data collectors. Data was collected after verbal consent by five trained data collectors through interviewer- administered face to face interviews in February 2018 using a door to door approach. Verbal consent is commonly used in Ethiopia due to the high illiteracy rate in rural region. The use of oral consent was discussed and recommended with the institutional review board of Addis Ababa University. The collection process was supervised by two trained supervisors. All data collectors were observed intermittently during the data collection process to ensure the quality of the interview. Before leaving a Kebele the questionnaires were checked for consistency and completeness. Incomplete questionnaires were taken back for re-interviewing.

### Methods of analysis

Incomplete questionnaires were excluded from all analysis. Descriptive and summary statistic was done for dependent and independent variables using SPSS 25. The variables marital status, occupation and ethnicity were summarized, and the household income was converted from Ethiopian Birr to USD, using the exchange rate of the February 26, 2018 (1 USD = 27.25 ETB). Independent variables were checked for multicollinearity using the Pearson correlation and chi square test. Some minor, but tolerable associations were found.

Sensitivity analysis was done using the Hosmer-Lemeshow test to analyze goodness of fit of the regression model. As a result, the Hosmer-Lemeshow test was 0.957 for the knowledge section, 0.903 for the attitude section and 0.00 for the practice section. The Practice section contained only 3 questions, so all analyses done to test for sensitivity might be inconclusive. Results of the logistic regression model to assess the practice of cervical cancer screening were included for their face validity. Logistic regression was used to create odds ratios in order to determine the strength of association in between independent and dependent variables using a level of significance of *p* < 0.05. Variables were included individually to select a robust model.

## Results

The response rate was 96.3% with 341 out of 354 women completing the questionnaire. 251 (73.6%) participants were Muslims, 64 (18.7%) Ethiopian Orthodox Christian, and 26 (7.6%) protestant Christians. The majority was married, housewife and lived in a rural setting. The mean age was 35.5 years (SD = 5.6 years). In February 2018 the mean household income was 31.95 $ (SD = 47.56 $). Most women had no formal education with a mean of 2.0 years (SD = 2.74 years). Only 9 women had further education after high school. In average the women had 4.4 children with a range of 0–12 children. (see Table 1).

**Table 1:** Socio- demographic information of participating women in Butajira, Ethiopia

Variable	Category	Frequency (n)	Relative frequency (%)
Religion (n = 341)	Muslim	251	73.6
	Not Muslim	90	26.4
Marital Status (n = 341)	Married	325	95.3
	Not Married	16	4.7
Occupation (n = 336)	Housewife	297	88.3
	Not Housewife^a^	39	11.7
Residence (n = 341)	Urban	34	9.7
	Rural	307	90.3
Education (n = 341)	No formal education	217	63.6
	Elementary school (1-8 yrs)	104	30.5
	Education beyond 9 years	11	3.3
	Higher education beyond high school	9	2.6
Household income per month in USD (n = 339)	< 10 USD	81	23.9
	10–50 USD	204	60.2
	50–100 USD	32	9.4
	> 100 USD	22	6.5
Use of contraceptives (n = 340)	yes	203	59.7
Current use of contraceptives (n = 341)	yes	83	24.3

^a^private employee 14, governmental employee 6, merchant 15, farmer/ daily labor 3, student 1

Only a third of the women had heard about cervical cancer and most were unable to name symptoms. Only few women correctly named screening as a method for reducing the risk of developing cervical cancer. None of the women named HPV as a risk factor. Commonly mentioned risk factors were smoking, HIV, multiple sexual partners, early sexual initiation, and STDs. 38 women correctly identified “middle” (30–49 years) as the age at risk of developing cervical cancer. The median of the score was 2 points out of 35 for the risk factor and general knowledge section combined. 139 (40.8%) were considered knowledgeable (see Table 2).

**Table 2:** Women’s knowledge on cervical cancer, screening, and risk factors in Butajira, Ethiopia

Variable	Yes n (%)	No n (%)	I don’t know n (%)
Heard of CC (n = 341)	125 (36.7)	2 (0.6)	214 (62.7)
Mentioned symptoms (n = 341)	14 (4.1)	7 (2.1)	320 (93.8)
*Bleeding*	14 (4.1)		
*Discharge*	2 (0.6)		
Risk reducing possible (n = 341)	19 (5.5)	7 (2.1)	315 (92.4)
Methods for risk reducing (n = 341)			
*Lifestyle*	3 (0.9)		
*Screening*	13 (3.8)		
Screening available in community (n = 340)	113 (33.1)	4 (1.2)	223 (65.4)
Screening methods (n = 340)			
VIA	0 (0)	(0)	340 (100)
*HPV test*	4 (1.2)	2 (0.6)	334 (97.9)
*Cytology*	3 (0.9)	1 (0.3)	336 (98.5)
Age at risk for CC (n = 341)			
*Young (< 30 yrs.)*	30 (8.8)		
*Middle (30–49 yrs.)*	38 (11.1)		
*Old (50–70 yrs.)*	9 (2.6)		
*Senile (> 70 yrs.)*	5 (1.5)		
*I don’t know*	271 (79.5)		
HPV as risk factor (n = 338)	**0 (0)**	1 (0.3)	337 (98.8)
HIV as risk factor (n = 341)	74 (21.7)	12 (3.5)	255 (74.8)
Multiple sexual partners as risk factor (n = 341)	86 (25.2)	6 (1.8)	249 (73.0)
Early sexual initiation as risk factor (n = 341)	82 (24.0)	11 (3.2)	248 (72.8)
History of STD as risk factor (n = 341)	84 (24.6)	4 (1.2)	253 (74.2)
Multi-parity as risk factor (n = 341)	68 (19.9)	31 (9.1)	242 (71.0)
Use of contraceptive as risk factor (n = 340)	40 (11.7)	23 (6.7)	277 (81.2)
Smoking as risk factor (n = 341)	110 (32.3)	5 (1.5)	226 (66.3)

Almost two third of the women thought cervical cancer was deadly and more than half stated it was a serious disease, but only 13.5% felt susceptible to cervical cancer. Half of the women thought screening was possible. Barriers were evaluated by asking for fear of screening procedure. A quarter of the women was scared of screening. For self-efficacy the proxy variables wish for screening, treatment possibilities and wish for treatment were selected. The majority of women wanted to know if they have cervical cancer. Most women thought cervical cancer was treatable and wanted to get treated, if they had cancer. For social acceptability the husband’s perspective towards screening and treatment as well as community and personal support were assessed. Women were asked to describe their husband’s perspective on screening and treatment of cervical cancer. The majority stated their husband would allow them to go for screening and treatment. 260 women (76.2%) would personally support women with cervical cancer and 230 (67.4%) said their community would be supportive of cervical cancer patients.

**Fig. 1:**
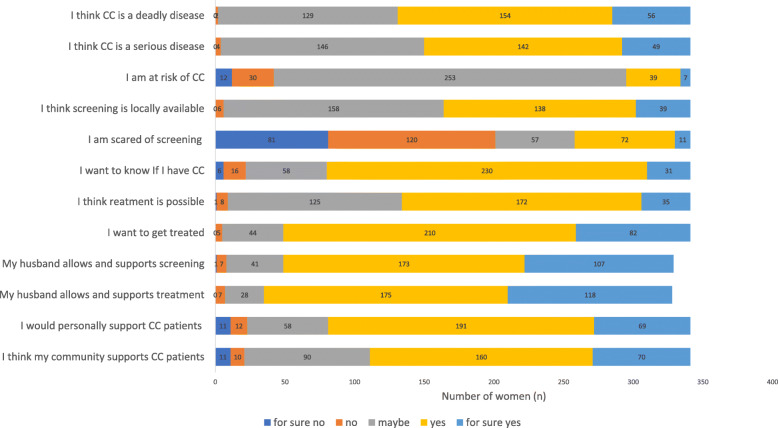
Women’s attitude towards cervical cancer and cervical cancer screening

The median of the attitude score was 8 points out of 12, so accordingly 202 (59.2%) women had a negative attitude towards cervical cancer and its screening (see Fig. 1).

Only eight women (2.3%) had been screened before. 240 women (70.4%) had the intention to be screened, however only 107 (31.4%) said they had access to a screening facility. The median of the practice score was one point out of three, so 102 (29.9%) women were considered to have good screening practice.

**Fig. 2:**
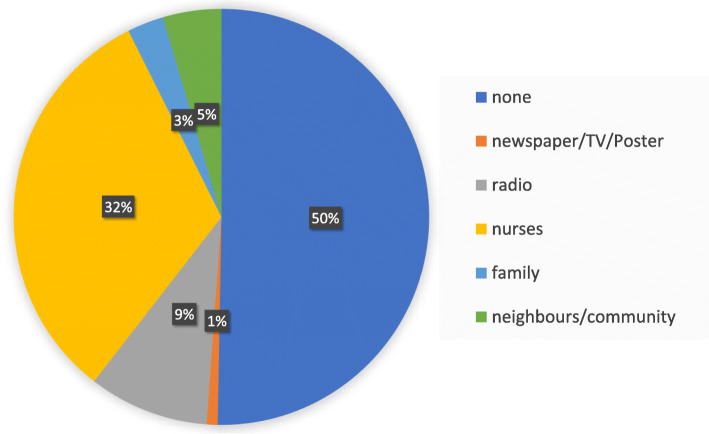
Women’s source of information concerning cervical cancer and cervical cancer screening

16 participants (4.7%) felt well informed about cervical cancer. Additionally, 300 (88%) answered they would like to learn more about it. Most women had no source of information (see Fig. 2).

Women with 1–8 years of education had 2.4 times the odds to be knowledgeable (CI:1.36–4.3) than those without any education. Women who had any source of information concerning cervical cancer were 9.1 times more likely to have good knowledge (CI:4.0–20.6), than those who had no source of information. Nurses as a source of information compared to those without information did not significantly raise the odds to be knowledgeable. Having used contraceptives before made it 2.3 times more likely for women to have good knowledge compared to those who never used contraceptives (CI: 1.3–4.1). Every additional dollar per month made it 1.009 times more likely for women to have good knowledge about cervical cancer (CI:1.00–1.01) (see Table 3).

**Table 3:** Factors associated with good knowledge of women in Butajira, Ethiopia

Variable	OR	95% CI for OR	*p*-value
Education (1–8 yrs. vs none)	2.42	1.36–4.30	**0.002**
Education (9 or more yrs. vs none)	2.30	0.67–7.82	0.18
Higher age	1.02	0.97–1.07	0.415
Source (nurse vs none)	1.52	0.86–2.66	0.143
Source (another source vs none)	9.10	4.00–20.66	**< 0.001**
Residence (urban vs rural)	0.79	0.29–2.15	0.646
Religion (not Muslim vs Muslim)	1.44	0.82–2.55	0.2
Occupation (any occupation vs housewife)	1.58	0.73–3.42	0.244
Contraceptive (ever used vs never used)	2.35	1.34–4.11	**0.003**
Household income per month (USD)	1.009	1.001–1.016	**0.024**

Women who named nurses as a source of information had 4.28 times the odds of having a positive attitude towards cervical cancer (CI:2.4–7.4) and women who named any other source of information had 5.06 times the odds of having a positive attitude (CI:2.4–10.3). Living in an urban setting made it 3.35 times more likely to have a positive attitude towards cervical cancer screening compared to women living in rural areas (CI:1.2–9.0). Women who ever used contraceptives had 2.2 the odds of having a positive attitude compared to those who never used contraceptives before (CI:1.2–3.8) (see Table 4).

**Table 4:** Factors associated with positive attitude towards screening of women in Butajira, Ethiopia

Variable	OR	95% CI for OR	*p*-value
Education (1–8 yrs. vs none)	1.39	0.78–2.47	0.264
Education (9 or more yrs. vs none)	1.24	0.38–4.00	0.718
Higher age	1.01	0.96–1.06	0.63
Source (nurse vs none)	4.28	2.46–7.43	**< 0.001**
Source (another source vs none)	5.06	2.48–10.33	**< 0.001**
Residence (urban vs rural)	3.35	1.23–9.07	**0.017**
Religion (not Muslim vs Muslim)	1.13	0.65–1.97	0.658
Occupation (any occupation vs housewife)	0.54	0.23–1.28	0.164
Contraceptive (ever used vs never used)	2.21	1.28–3.84	**0.004**
Household income per month (USD)	1.001	0.995–1.007	0.747

Women who named nurses as a source of information had 21.05 times the odds to have a good practice score than those who named no source (CI: 10.4–42.3) and women who named any source of information had 5.8 times the odds to have a good practice score than those who had no source of information (CI:2.4–13.5) (see Table 5).

**Table 5:** Factors associated with good practice of women in Butajira, Ethiopia

Variable	OR	95% CI for OR	*P*-value
Education (1–8 yrs. vs none)	1.66	0.83–3.29	0.147
Education (9 or more yrs. vs none)	0.66	0.16–2.67	0.563
Higher age	1.00	0.94–1.06	0.883
Source (nurse vs none)	21.05	10.47–42.34	**< 0.001**
Source (another source vs none)	5.82	2.49–13.59	**< 0.001**
Residence (urban vs rural)	1.02	0.32–3.27	0.962
Religion (not Muslim vs Muslim)	0.57	0.29–1.11	0.099
Occupation (any occupation vs housewife)	0.29	0.08–1.103	0.07
Contraceptive (ever used vs never used)	0.92	0.48–1.76	0.814
Household income per month (USD)	0.99	0.99–1.00	0.855

## Discussion

As one of the few community-based studies conducted in rural Ethiopia with a door to door approach, external validity can be considered high [31]. In comparison to studies conducted in Ethiopia and other African countries awareness of cervical cancer was low in Butajira, since only a third of the participants had heard about the disease before. Other Ethiopian studies conducted in an urban setting reported a higher level of awareness; in Dessi town, 57.7% of the study population were aware of cervical cancer [27], in Mekelle, 85% [3], in Gondar 78.7% [23] and in Addis Ababa 50% [14]. Lack of awareness has proven to be one of the major barriers towards cervical cancer screening [10]. In a qualitative study conducted in Burkina Faso it was mentioned as the second most common reason for underutilization [32].

57.8% of the population in Butajira did not know any risk factors. This result was similar to those in Dessie town, Ethiopia (58.1%), in Gondar, Ethiopia (47.5%) and in Hossana, Ethiopa (58.3%). None of the participants in Butajira identified HPV as a risk factor of cervical cancer. In Hossana, Ethiopia 8.9% named HPV as a risk factors and in Kenya 16.9% [33]. Awareness and knowledge of HPV as a risk factor is becoming increasingly important, as HPV vaccine campaigns and HPV-based screening methods are scaled up in many countries and is also part of the guideline for cervical cancer prevention and control in Ethiopia [21, 34, 35]. The Ethiopian government included raising awareness about cancer related infections such as HPV in the national program 2015 [20, 21] In contrast to other studies, in which participants most commonly named multiple sexual partners [9, 27, 33],or STDs [23], the most commonly named risk factor in Butajira was smoking (110; 32.3%). Perception of risk factors like smoking, HIV, multiple sexual partners, and history of STD might be biased by a generally negative attitude against them. Women who used contraception were more knowledgeable and had a better attitude towards cervical cancer screening, than those who did not. Similar results have been found in rural Kenya [36] and Uganda [37] and could be explained by the contact to medical care and better health seeking behavior.

Only 13.5% of the participants felt at risk of developing cervical cancer. In Finote Selam, Ethiopia, 51.5% of the women felt at risk [24], and in Uganda 76% [38]. In Hossana, Ethiopia, 54% of the participants stated cervical cancer was curable [9], which is comparable to Butajira, where 60.7% said cervical cancer was treatable. In contrast to many studies, women in Butajira felt supported by their husbands to go for screening (82.1%) and for treatment (85.9%), while in Kenya many women mentioned fear of marital dispute and commonly did not feel supported by their partners [39], the same was recorded in Uganda [37]. In the qualitative study conducted in Jimma, Ethiopia and Addis Ababa, Ethiopia, women also named fear of divorce and shame as one of the major barriers to cervical cancer screening utilization [12].

In Butajira only 2.3% of the women had been screened before. There was no existing cervical cancer screening program in Butajira at the time of data collection. This is less than found in other Ethiopian studies conducted in urban areas such as Hossana (9.9%) [9], and in Yirgalem (9.2%) [18], but higher than the average screening rate in Ethiopia of 0.6% [13]. Studies in Ethiopia focusing on HIV positive women revealed higher screening rates of 11.5% [13].

Health professionals were the most commonly named source of information. Participants in Hossana, Ethiopia also mostly named health professionals or media as a source [9, 14]. Studies conducted in Ethiopia revealed misconceptions about causes, risk factors, risk reduction and screening among health workers [4, 40]. Only 11.4% [4] and 22% [40] of the female health workers had been screened for cervical cancer. Appropriately informed nurses can inspire women to utilize offered cervical cancer screening programs [41]. Surprisingly, naming nurses as a source of information was not statistically significantly associated with a better outcome of the knowledge score. This can raise questions about the health education provided by health workers among the communities. However, naming nurses as source of information was positively associated with the attitude and practice score, putting further emphasis on the relevance of their education. Since brochures, posters and newspapers together were named by 0.6% only, campaigns should focus on oral information distribution. Religion has been mentioned as a source of information by 1.6% of participants in a study conducted in southern Ethiopia among health workers [4], but has not been mentioned as a source of information in Butajira. In contrast to other African studies communication about cervical cancer seems low in the communities in Butajira, since only 2.9% named family and 4.1% neighbors as a source of information. In a qualitative study in Uganda, participants mostly named their aunts and elders within the community as a source of information [37] and in Congo most participants said they heard about cervical cancer from conversations with other people [25]. Having sufficient information on cervical cancer has been linked with better uptake of screening procedures [42]. This further proves the need for accurate awareness campaigns concerning cervical cancer.

Many other studies in Ethiopia have been conducted in urban areas, providing better access to health care and information. The urban setting of Butajira was also associated with a higher attitude score, possibly due to better access to information and health care.

Findings from this study were used to develop appropriate sensitization material and identify possible barriers for the following study on adherence to screening [17]. Furthermore, women who had previously been screened were not included in the upcoming trial, therefore defining the screening rate in Butajira was an important part of this study. Health extension workers were used for community mobilization during the trial. Since naming health workers as a source of information was not statistically significantly associated to a better outcome on the knowledge score, special emphasis was put on their training to ensure the accuracy during the upcoming trial.

Several limitations have to be named concerning the study conducted in Butajira, Ethiopia. There is no standardized tool to assess knowledge, attitude and practice concerning cervical cancer and its prevention, therefore the comparability is limited. The study population was relatively homogenous in respect to residential area, religion and occupation. There are several limitations to knowledge, attitude and practice studies, as people might give socially desired answers [25] and sensitive subjects might not be answered correctly. Despite careful clustering, selection bias was possible, since participants absent during the time of data collection and those who did not want to participate were not included; this could affect the internal validity of the study [25].

## Conclusion

Awareness and knowledge of cervical cancer prevention and risk factors, especially HPV, was low in Butajira, rural Ethiopia. Women’s sense of low susceptibility towards cervical cancer was often not favorable for screening practice. Focus should be put on distributing information on risk factors, screening methods and their availability within the area of Butajira, Ethiopia. A higher level of education, having sources of information concerning cervical cancer and use of contraceptives were the most relevant socio-demographic factors for a positive outcome of knowledge, attitude and practice on regression analysis. Special emphasis should be put on training health care providers extensively on cervical cancer and its screening, since they are the primary source of information among the population in Butajira.

## Supplementary information

**Additional file 1. **Questionnaire for women. Questionnaire used within this study.

## Data Availability

The datasets used and analyzed during the current study are not publicly available due to data privacy of participants but are available from the corresponding author on reasonable request.

## Abbreviations

CC: Cervical cancer
CI: Confidence interval
HDSS: Health development surveillance site
HIV: Human immunodeficiency virus
HPV: Human papilloma Virus
KAP: Knowledge, attitude and practice
OR: Odds ratio
SD: Standard deviation
STD: Sexually transmitted disease
TBC: Tuberculosis
VIA: Visual inspection with acidic acid
WHO: World health organization
YRS: Years
