# Supplementary material for: Cervical cancer screening in rural Ethiopia: a cross- sectional knowledge, attitude and practice study
Source: BMC Cancer. 2020 Jun 17;20:563. doi: 10.1186/s12885-020-07060-4 (PMC7298871; doi:10.1186/s12885-020-07060-4)
Supplement: Supplementary file 1 — Additional file 1. Questionnaire for women. Questionnaire used within this study. [file 12885_2020_7060_MOESM1_ESM.pdf]

Knowledge, attitude and practice of cervical cancer/ reproductive cancer and its screening : a cross-sectional study

Questionnaire for women, version 06.02.2018

Questionnaire number:

HDSS Number:

Name of interviewer:

Date of interview:

District:

Kebele:

Start time:

Finish time:

Part 1: socio demographic characteristics of the respondents

Number	questions	answers
101	what is your age?	…years
102	What is your Religion?	1.orthodox<br>2.muslim<br>3.protestant<br>4.catholic<br>5.other
103	what is your marital status?	1.single<br>2.married<br>3.divorced<br>4.widowed<br>5.separated
104	what is your ethnicity?	1.gurage<br>2.silite<br>3.hadiya<br>4.kambata<br>5oromo<br>6.amhara<br>7.Tigire<br>8.other
105	what is the level of school you attended?	…years<br>higher education

Number	questions	answers
**106**	what is your occupation?	1.housewife<br>2.private employee<br>3.farmer<br>4.government employee<br>6.daily laborer<br>7.merchant<br>8.student<br>9.others
**107**	what is your current residence area?	1.city<br>2.village
**108**	do you have a monthly income?	1.yes<br>2. no<br>**If no, skip question 109**
**109**	how much money do you have this month?	**..**birr
**110**	do you have health insurance?	1.yes<br>2.no

Part 2: respondents reproductive data

number	questions	answers
**201**	have you ever been pregnant?	1.yes<br>2.no<br>**if no, skip question 202**
**202**	if yes how many times have you been pregnant?	…times
**203**	have you ever given birth?	1.yes<br>2.no<br>**if no, skip question 204 and 205**
**204**	how old where you when you first gave birth?	…years
**205**	how many children do you have?	…
**206**	how old were you when you first had sexual intercourse?	1. …years<br>2. I don't know<br>3. Never
**207**	did you use any contraceptive methods	1.yes<br>2.no
**208**	are you currently using contraceptive methods?	1.yes<br>2.no<br>**if no, skip question 209**

number	questions	answers
**209**	if yes which method are you currently using?	1.IUD<br>2.injectable<br>3.implants<br>4.pills<br>5.male condom<br>6.female condom<br>7.others

Part 3 : general knowledge about cervical cancer/ reproductive cancer

number	question	answer
**301**	have you ever heard of cervical cancer/ reproductive cancer?	1.yes<br>2. no<br>3. I don't know
**302**	Have you ever had contact with someone with cervical cancer/ reproductive cancer?	1.yes<br>2.no<br>3. I don't know
**303**	Do you know symptoms of cervical cancer/ reproductive cancer?	1.yes<br>2. no<br>3. I don't know<br>**if no/I don't know skip question 304**
**304**	which symptoms do you know?	1.abnormal bleeding<br>2.discharge<br>3.pelvic pain<br>4.bleeding after sex<br>**Participant can name more than one answer**
**305**	do you think that there is a way to reduce the risk of developing cancer?	1.yes<br>2. no<br>3. I don't know<br>**if no/ I don't know, skip questi-on 306**
**306**	if yes how can you reduce your risk?	1.modifying lifestyle<br>2.vaccine<br>3.screening<br>4.use of condom<br>**Participant can name more than one answer**
**307**	Can you get vaccinated against cervical cancer/ reproductive cancer?	1.yes<br>2. no<br>3. I don't know
**308**	is it possible to screen for cervical cancer/ reproductive cancer?	1. yes<br>2. no<br>3. I don't know

number	question	answer
**309**	do you know any women that have been screened?	1. yes<br>2. no<br>3. I don’t know
**310**	Do you know any method of screening?	1. yes<br>2. no<br>3. I don’t know
**311**	have you heard of VIA?	1. yes<br>2. no<br>3. I don’t know<br>**if no/ I don’t know, skip questi-on 315**
**312**	how often should a women go for VIA	1.every year<br>2.every 3 year<br>3.every 5 years<br>4.every 10 years<br>5.only once<br>**Please take down only one an-swer**
**313**	have you heard of HPV test?	1. yes<br>2. no<br>3. I don’t know
**314**	have you heard of the cytological examination?	1.yes<br>2. no<br>3. I don’t know
**315**	do you think early detection is helpful?	1. yes<br>2. no<br>3. I don’t know

Part 4: participant’s perception of risk factors of cervical cancer/ reproductive cancer

number	question	responses
**401**	Which age group has the highest risk of developing cervical cancer/ reproductive cancer?	1. 15-30<br>2. 30-49<br>3. 50-70<br>4. >70<br>5. I don’t know<br>**participant can name more than one answer**
**402**	Is HPV infection a risk factor for cervical cancer/ reproductive cancer?	1. yes<br>2. no<br>3. I don’t know
**403**	Is HIV infection a risk factor for cervical cancer/ reproductive cancer?	1. yes<br>2. no<br>3. I don’t know

number	question	responses
**404**	Is having multiple sexual partners a risk factor for cervical cancer/ reproductive cancer?	1.yes<br>2. no<br>3. I don’t know
**405**	Is early sexual intercourse a risk factor for cervical cancer/ reproductive cancer?	1.yes<br>2. no<br>3. I don’t know
**406**	Is a history of sexually transmitted diseases a risk factor for cervical cancer/ reproductive cancer?	1.yes<br>2. no<br>3. I don’t know
**407**	Are a large number of pregnancies and childbirths risks factors for cervical cancer/ reproductive cancer?	1.yes<br>2. no<br>3. I don’t know
**408**	Is hormonal contraception a risk factor?	1.yes<br>2. no<br>3. I don’t know
**409**	is occurrence of cervical cancer/ reproductive cancer in the family a risk factor?	1.yes<br>2. no<br>3. I don’t know
**410**	Is smoking a risk factor?	1.yes<br>2. no<br>3. I don’t know

Part 5: attitude towards the disease and it’s screening

number	question	answer
**501**	Cervical cancer/ reproductive cancer is a serious disease and can be deadly.	1<br>2<br>3<br>4<br>5
**502**	Cervical cancer/ reproductive cancer is a serious disease in my community	1<br>2<br>3<br>4<br>5
**503**	I can get cervical cancer/ reproductive cancer.	1<br>2<br>3<br>4<br>5
**504**	It is possible to screen for cervical cancer/ reproductive cancer.	1<br>2<br>3<br>4<br>5

number	question	answer
**505**	I am scared of the screening process	1<br>2<br>3<br>4<br>5
**506**	I want to know if I have cervical cancer/ reproductive cancer.	1<br>2<br>3<br>4<br>5
**507**	Cervical cancer/ reproductive cancer can be treated.	1<br>2<br>3<br>4<br>5
**508**	I want to get treated, if I have cervical cancer/ reproductive cancer.	1<br>2<br>3<br>4<br>5
**509**	My husband allows me/ supports me to get tested for cervical can-cer/ reproductive cancer.<br><br>**Skip question 509 and 510, if the woman is not married**	1<br>2<br>3<br>4<br>5
**5010**	My husband allows me/ wants me to get treated if I have cervical cancer/ reproductive cancer.	1<br>2<br>3<br>4<br>5
**511**	I feel supportive and positive towards people with cervical cancer/ reproductive cancer in your community	1<br>2<br>3<br>4<br>5
**512**	My community feels supportive and caring for people with cervical cancer/ reproductive cancer.	1<br>2<br>3<br>4<br>5

likert scale
1 for sure no
2 no
3 maybe
4 yes
5 for sure yes

Part 6: practice of cervical cancer/ reproductive cancer screening

number	question	answer
**601**	Have you ever been screened for cervical cancer/ reproductive cancer?	1.yes<br>2.no
**602**	Do you want to get screened for cervical cancer/ reproductive cancer?	1.yes<br>2.no
**603**	Do you have access to cervical cancer/ reproductive cancer screening?	1.yes<br>2.no

Part 7: sources of information

number		
**701**	Do you feel well informed about cervical cancer/ reproductive cancer?	1.yes<br>2.no
**702**	Do you want to lern more about cervical cancer/ reproductive cancer?	1.yes<br>2.no
**703**	What is your source of know-ledge?<br><br>**Participant can name more than one answer**	1.none<br>2.newspaper/magazine<br>3.Radio<br>4.Television<br>5.Billboards, posters<br>6.brochures<br>7.health workers<br>8.family, friends,<br>9.neighbors, colleges<br>10.religious leaders<br>11.teachers<br>12.others
